# IV–Oral Antibiotic Switch in Streptococcal and Enterococcal Endocarditis: Evaluating ESC Versus WikiGuideline Criteria

**DOI:** 10.3390/pathogens15090895

**Published:** 2026-08-25

**Authors:** Anna Mohamad, Frederik M. A. van den Heuvel, Ilse J. E. Kouijzer

**Affiliations:** 1 Department of Internal Medicine, Radboud University Medical Center, 6500 HB Nijmegen, The Netherlands; 2 Department of Cardiology, Radboud University Medical Center, 6500 HB Nijmegen, The Netherlands; frederik.vandenheuvel@radboudumc.nl; 3 Radboud Community for Infectious Diseases, Radboud University Medical Center, 6500 HB Nijmegen, The Netherlands

**Keywords:** endocarditis, IV–oral switch, guidelines, oral antibiotic treatment

## Abstract

**Background:** The 2023 European Society of Cardiology (ESC) guideline and the 2023 WikiGuideline both support intravenous (IV)–oral antibiotic switch in selected patients with infective endocarditis but apply different eligibility criteria. We aimed to assess hypothetical eligibility according to both guideline sets and to describe actual IV–oral switch practice at our center. **Methods:** In this retrospective observational study, adult patients with possible or definite streptococcal or enterococcal infective endocarditis treated between January 2024 and January 2026 were included. Eligibility for IV–oral switch was retrospectively assessed according to the original ESC criteria and WikiGuideline criteria using a predefined hypothetical switch date. Actual IV–oral switches performed in clinical practice and their clinical outcomes were described separately. **Results:** Ninety patients were included. Based on the original ESC criteria, two patients (2.2%) were hypothetically eligible for IV–oral switch, compared with 67 patients (74.4%) according to the WikiGuideline criteria. The principal reason for ESC ineligibility was the absence of repeat transesophageal echocardiography before the hypothetical switch date. In clinical practice, 22 patients (24.4%) underwent IV–oral switch. None of these patients fulfilled original ESC eligibility criteria, whereas all fulfilled the WikiGuideline criteria. Clinical outcomes of patients who underwent IV–oral switch are presented descriptively. **Conclusions:** In this retrospective cohort, hypothetical eligibility for IV–oral switch differed substantially depending on whether the ESC or WikiGuideline criteria were applied. At our center, local imaging practice accounted for most ESC ineligibility. Prospective studies are needed to determine which eligibility criteria and imaging approaches best identify patients who can safely undergo IV–oral switch.

## 1. Introduction

Until recently, treatment for infective endocarditis required 4 to 6 weeks of intravenous (IV) antibiotic treatment [1]. Recent guidelines state that specific patients can safely switch to oral antibiotic treatment under certain conditions [2,3], which can reduce healthcare costs and catheter-related complications [4].

The POET trial demonstrated that IV–oral switch in stable patients with left-sided infective endocarditis after at least 10 days of IV treatment is safe and effective [4]. A post hoc analysis of five-year outcomes found oral therapy non-inferior to IV, with lower all-cause mortality in the IV–oral switch group [5]. The POETry study confirmed these findings, showing lower mortality and shorter hospital stay in patients after IV–oral switch [6].

After the POET trial, the European Society of Cardiology included IV–oral switch criteria in their endocarditis guidelines [3]. The WikiGuideline for endocarditis from 2023 has also incorporated IV–oral switch criteria for endocarditis patients [2].

The objectives of this retrospective study were to (1) determine hypothetical eligibility for IV–oral switch according to the original ESC criteria, (2) determine hypothetical eligibility according to the WikiGuideline criteria, and (3) describe actual IV–oral switch practice in a cohort of patients with streptococcal and enterococcal infective endocarditis.

## 2. Material and Methods

In this retrospective, observational study, adult patients with possible or definite endocarditis according to the modified Duke criteria [3] and treated at Radboudumc and regional hospitals between 1 January 2024 and 1 January 2026 were included. Possible endocarditis patients who were treated for endocarditis were also included as the diagnosis of endocarditis is not always clear, reflecting routine clinical practice. Only patients with streptococcal or enterococcal endocarditis were included because these pathogens represent the group with the strongest evidence supporting IV–oral switch. Patients with rejected endocarditis were excluded. Cases were identified via CTcue (data extraction software, version 4.13 ) and verified with additional clinical data from Radboudumc medical records.

Treatment details such as start date and type and duration of antibiotic treatment were recorded, excluding empirical therapy. Outcomes evaluated three months post-treatment included all-cause mortality, relapse, reinfection, readmission and reoperation. Relapse was defined as recurrence due to the same pathogen, and reinfection as a new episode with a different pathogen. Readmission was categorized as related or unrelated to endocarditis and reoperation as either directly or indirectly linked to endocarditis.

A hypothetical IV–oral switch date was set at two weeks after the start of targeted IV therapy, ensuring at least 10 days of IV therapy, in line with ESC guidelines. If cardiac surgery was performed before this date, the switch date was postponed to 7 days post-surgery, as recommended [3]. The ESC and WikiGuideline criteria for IV–oral switch were assessed based on this hypothetical date and are illustrated in Table 1 [2,3]. Patients with simultaneous right- and left-sided endocarditis were classified as left-sided [4].

Suppressive therapy, antibiotic switch due to side effects, and oral treatment for endocarditis-related complications were not considered actual IV–oral switches. According to the ESC guidelines, a transesophageal echocardiogram (TEE) within 48 h was required. Eligibility according to the ESC guideline was assessed using the original ESC criteria [3]. In addition, a post hoc sensitivity analysis was performed in which a transthoracic echocardiogram (TTE) within 7 days before the hypothetical switch date was accepted as a center-specific alternative to TEE.

For the WikiGuideline analysis, eligibility was assessed according to the original WikiGuideline criteria [2]. For both guideline analyses, the criterion “other indications for continuing IV therapy” was operationalized using the ESC-defined contraindications (vascular graft involvement, CIED infection without device removal, epidural abscesses, and perivalvular complications), as in our study there were no socioeconomic determinants of health as described in the WikiGuideline. Decisions regarding actual IV–oral switch in clinical practice were made by the treating multidisciplinary endocarditis team as part of routine care and were not protocolized or prospectively recorded.

The primary outcome was the number of patients with endocarditis who were hypothetically eligible for an IV–oral switch according to the ESC guideline and the WikiGuideline. Secondary outcomes were the number of patients who underwent IV–oral switch in clinical practice and the clinical outcomes of these patients. Descriptive statistics (frequencies (%) and medians [IQR]) were used in this study, and analyses were performed in SPSS 29.0.

## 3. Results

**Figure 1:**
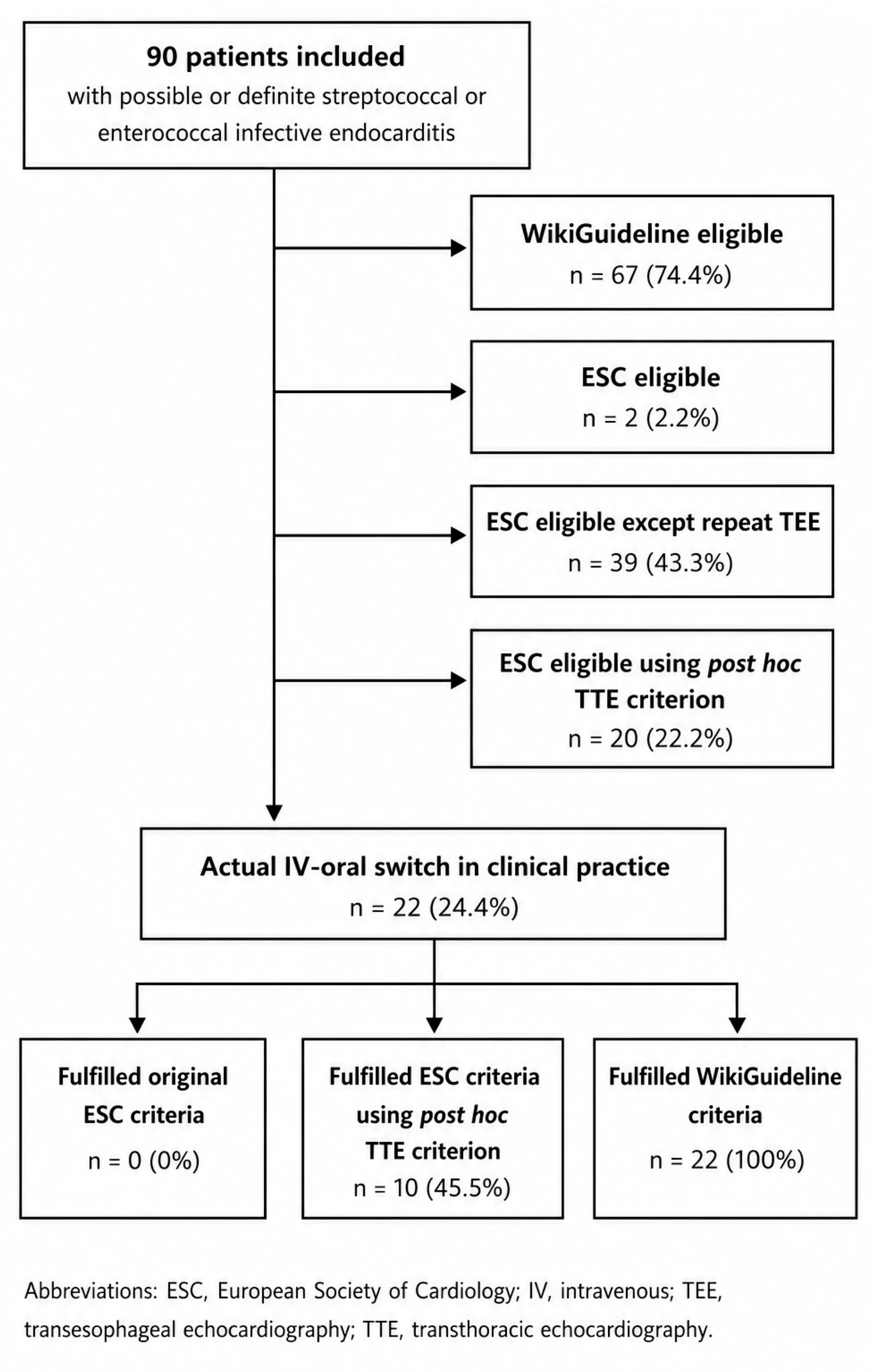
Flow diagram of hypothetical eligibility for IV–oral switch according to the ESC guideline and WikiGuideline, and actual IV–oral switch in clinical practice.

Between January 2024 and January 2026, a total of 90 patients with streptococcal or enterococcal endocarditis were included for analysis (Table 2). Patient inclusion, hypothetical eligibility according to the ESC guideline and WikiGuideline, and actual IV–oral switch in clinical practice are summarized in Figure 1. Of all patients, 22 patients (24.4%) underwent IV–oral switch in clinical practice, whereas 68 patients (75.6%) were treated with prolonged IV therapy.

According to the ESC guideline, only two out of 90 patients (2.2%) were hypothetically eligible for an IV–oral switch. In contrast, when applying the WikiGuidelines, 67 patients (74.4%) were hypothetically eligible for IV–oral switch. A total of 39 patients fulfilled all original ESC criteria except the requirement for repeat TEE, indicating that repeat imaging was the principal determinant of ESC ineligibility. According to the ESC switch criteria, only six patients (6.7%) underwent a TEE 2 days prior to the hypothetical switch date, as shown in Table 3. In a post hoc sensitivity analysis, accepting a TTE within 7 days before the hypothetical switch date increased the number of hypothetically eligible patients according to the ESC criteria from 2 to 20 (22.2%). Although two patients were eligible for IV–oral switch according to the ESC criteria, both patients did not switch to oral antibiotic treatment in clinical practice.

A total of 22 patients underwent IV–oral switch in clinical practice: 18 patients (81.8%) with streptococcal endocarditis and four patients (18.2%) with enterococcal endocarditis. Of the 18 patients with streptococcal endocarditis, 17 patients were initially treated with benzylpenicillin IV and one was treated with benzylpencillin IV in combination with gentamicin. After IV–oral switch, 17 were switched to amoxicillin orally and one was switched to oral clindamycin. In enterococcal endocarditis, all four patients were initially treated with amoxicillin and ceftriaxone; after IV–oral switch, three were switched to linezolid and one to amoxicillin orally. The median time until switch was 16 days (IQR 10.5–32). All patients who underwent IV–oral switch fulfilled the WikiGuideline criteria, whereas none fulfilled the original ESC criteria. Ineligibility was due to absence of TEE in all patients, but was also due to right-sided endocarditis (n = 2), insufficient decline of C-reactive protein (n = 3), body mass index > 40 (n = 1) and persistent fever (n = 1). Using the post hoc TTE criterion, 10 of the 22 patients who underwent IV–oral switch fulfilled the modified ESC criteria. The clinical outcomes of patients who underwent IV–oral switch are summarized in Table 2. One patient in the IV–oral switch group was readmitted after successful treatment for streptococcal endocarditis. The reason for readmission was a *Klebsiella* bacteremia, and this patient died during readmission due to decompensated liver cirrhosis. In one patient with *E. faecalis* CIED infection, IV–oral switch (linezolid) was performed because of toxicity on IV treatment although the CIED was not removed. No side effects were seen in the patients who were on oral antibiotic treatment.

## 4. Discussion

This is the first study to compare eligibility for IV–oral switch according to the 2023 ESC guideline and the 2023 WikiGuideline in patients with infective endocarditis. Our study demonstrates substantial differences in hypothetical eligibility depending on which guideline is applied. According to the original ESC criteria, only two patients (2.2%) were hypothetically eligible for IV–oral switch compared with 67 (74.4%) according to the WikiGuideline. The main reason for the low ESC eligibility was local practice: at our center, a TEE is not routinely performed before switch, while ESC guidelines require a negative TEE within two days before IV–oral switch. The requirement for a TEE within two days before IV–oral switch had a major impact on eligibility for IV–oral switch in our cohort. In a post hoc sensitivity analysis, accepting a recent TTE substantially increased the number of patients fulfilling the ESC criteria. For the Dutch setting, a TTE with adequate quality has been accepted as an alternative for TEE in considering IV–oral switch in endocarditis [7]. Prospective studies are needed to determine which imaging strategy best identifies patients suitable for IV–oral switch.

Twenty-two patients underwent IV–oral switch in routine clinical practice. Because treatment allocation was not randomized and patients were selected for oral therapy based on clinical judgment, the clinical outcomes of these patients should be interpreted as descriptive findings. Similar retrospective studies found no significant differences in mortality, relapse or readmission between oral and IV groups, supporting the feasibility of oral therapy in clinically stable patients [8,9,10,11]. The ENDO-ORAL study also compared patients who did or did not meet eligibility criteria according to the POET trial and found that outcomes were similar in both groups [11]. Interestingly, over half of both IV and oral patients met POET eligibility, suggesting that many IV patients could have been switched, while some oral patients were safely switched despite not fulfilling criteria. Notably, ENDO-ORAL did not account for infection parameters and did not apply the ESC requirement of a TEE within two days, which may explain their higher eligibility rates compared with our study.

The substantial difference in hypothetical eligibility between the ESC guideline and the WikiGuideline reflects differences in the eligibility criteria applied by both recommendations, which are partly rooted in their underlying evidence base. The ESC guideline recommendations for IV–oral switch are primarily derived from the POET trial, whereas the WikiGuideline incorporates the totality of published evidence on oral step-down therapy for infective endocarditis. In our cohort, the requirement for repeat TEE before IV–oral switch accounted for most ESC ineligibility, illustrating how specific eligibility criteria and local implementation of guideline recommendations, particularly imaging practice, may substantially influence the proportion of patients considered eligible for IV–oral switch. These findings underline the importance of a multidisciplinary approach to the assessment of patients for IV–oral switch. Whether differences in eligibility criteria translate into clinically meaningful differences in patient outcomes remains unknown and requires prospective evaluation.

Limitations of this study included the retrospective design and its risk for selection bias, restriction to streptococcal and enterococcal endocarditis, partly incomplete follow-up from regional centers and the small number of IV–oral switches. After the implementation of IV–oral switch in the endocarditis guidelines, our center started IV–oral switch in endocarditis patients in 2024. Because experience with IV–oral switch was still evolving, not all patients who may have been eligible underwent oral treatment.

In conclusion, our study demonstrates substantial differences in hypothetical eligibility for IV–oral switch according to the ESC guideline and the WikiGuideline. In our cohort, local imaging practice accounted for most ESC ineligibility. Prospective studies are needed to determine which eligibility criteria and imaging approaches best identify patients who can safely undergo IV–oral switch.

## Figures and Tables

**Table 1:** ESC and WikiGuideline criteria for IV–oral switch.

	ESC Guideline	WikiGuideline
Location	Left-sided endocarditis	-
Micro-organism	Streptococci, *S. aureus*, *E. faecalis*, CNS	-
Clinical course	Fever free > 2 days CRP < 25% of peak value or CRP < 20 mg/L Leukocytes < 15 × 10^9^/L.	Clinically stable, no indication for further source control
Bacteremia	Adequate antibiotics for >10 days and >7 days after surgery	Intravenous treatment has cleared bacteremia
TEE	Transesophageal echocardiogram < 2 days before switch and no indication for abscess or surgery	-
Oral antibiotic regimen	Pathogen is sensitive for at least 2 antibiotics	Oral antibiotics available, pathogen is sensitive to treatment
Absorption	No gastro-intestinal uptake problems BMI < 40	No gastro-intestinal uptake problems
Other	No other indication for continuing intravenous antibiotics	No other indication for continuing intravenous antibiotics, including socioeconomic determinants

ESC: European Society of Cardiology; *S. aureus*: *Staphylococcus aureus*; *E. faecalis*: *Enterococcus faecalis*; CNS: coagulase negative staphylococcus; CRP: C-reactive protein; TEE: transesophageal echocardiogram; BMI: body mass index.

**Table 2:** Baseline characteristics and outcomes of IV-only group and IV–oral switch group.

	IV Only, n = 68	IV–Oral, n = 22	Total, n = 90
Characteristics			
Gender of patient, male n (%)	50 (73.5%)	18 (81.8%)	68 (75.6%)
Age, years, median (IQR)	71 (62.3–77.0)	69.5 (60.5–75.0)	71 (62.0–77.0)
BMI, median (IQR)	25.6 (22.0–27.9)	23.2 (21.1–26.6)	25.2 (21.7–27.8)
Medical history			
Charlson Comorbidity index, median (IQR)	3.0 (2.0–5.0)	3.0 (2.0–4.3)	3.0 (2.0–5.0)
History of endocarditis, n (%)	11 (16.2%)	1 (4.5%)	12 (13.3%)
Congenital heart disease, n (%)	8 (11.8%)	3 (13.6%)	11 (12.2%)
Native valve disease, n (%) ^a^	16 (30.8%)	4 (20%)	20 (27.8%)
Prosthetic valve, n (%)	29 (42.6%)	8 (36.4%)	37 (41.1%)
CIED, n (%)	17 (25.0%)	0 (0%)	17 (18.9%)
Duke definition			
Definite endocarditis, n (%)	62 (91.2%)	20 (90.9%)	82 (91.1%)
Valve affected			
Aortic valve	29 (42.6%)	11 (50.0%)	40 (44.4%)
Mitral valve	18 (26.5%)	7 (31.8%)	25 (27.8%)
Pulmonary valve	5 (7.4%)	2 (9.1%)	7 (7.8%)
Tricuspid valve	0 (0%)	0 (0%)	0 (0%)
Aortic valve and mitral valve	5 (7.4%)	1 (4.5%)	6 (6.7%)
Lead infection	6 (8.8%)	1 (4.5%)	7 (7.8%)
Aortic valve and lead infection	1 (1.5%)	0 (0%)	1 (1.1%)
Bentall	2 (2.9%)	0 (0%)	2 (2.2%)
Missing	2 (2.9%)	0 (0%)	2 (2.2%)
Duration of bacteremia in days ^b^, median (IQR)	3.0 (2.0–4.0)	2.0 (2.0–4.0)	2.5 (2.0–4.0)
Pathogen			
Streptococci, n (%)	49 (72.1%)	18 (81.8%)	67 (74.4%)
*S. bovis*	8 (11.8%)	1 (4.5%)	9 (10.0%)
*S. salivarius*	3 (4.4%)	0 (0%)	3 (3.3%)
*S. mutans*	2 (2.9%)	5 (22.7%)	7 (7.8%)
*S. mitis*	18 (26.5%)	4 (18.2%)	22 (24.4%)
*S. anginosus*	4 (5.9%)	0 (0%)	4 (4.4%)
Hemolytic group A	2 (2.9%)	0 (0%)	2 (2.2%)
Hemolytic group B	2 (2.9%)	0 (0%)	2 (2.2%)
Hemolytic group C + G	3 (4.4%)	1 (4.5%)	4 (4.4%)
*S. pneumoniae*	1 (1.5%)	1 (4.5%)	2 (2.2%)
*S. viridans*	6 (8.8%)	5 (22.7%)	11 (12.2%)
Other	0 (0.0%)	1 (4.5%)	1 (1.1%)
Enterococci, n (%)	19 (27.9%)	4 (18.2%)	23 (25.6%)
*E. faecalis*	15 (22.1%)	3 (13.6%)	18 (20.0%)
*E. faecium*	3 (4.4%)	1 (4.5%)	4 (4.4%)
Other	1 (1.5%)	0 (0%)	1 (1.1%)
Outcomes			
Mortality	5 (7.5%)	1 (4.5%)	6 (6.7%)
Readmission ^c^	14 (32.5%)	5 (22.7%)	19 (29.2%)
Endocarditis admission	5 (11.6%)	0 (0%)	5 (7.7%)
Admission other reason	9 (20.9%)	5 (22.7%)	14 (21.5%)
Reinfection	5 (7.7%)	1 (4.5%)	6 (6.9%)
Relapse	5 (7.6%)	0 (0%)	5 (5.7%)
Reoperation	4 (6.0%)	2 (9.0%)	6 (6.7%)
Reoperation due to complications after surgery	3 (4.5%)	1 (4.5%)	4 (4.5%)
Reoperation due to endocarditis	1 (1.5%)	1 (4.5%)	2 (2.2%)
Side effects of IV treatment ^d^	4 (7.4%)	5 (22.6%)	9 (11.8%)
Renal	4 (7.4%)	3 (13.6%)	7 (9.2%)
Gastro-intestinal	0 (0%)	0 (0%)	0 (0%)
Allergy	0 (0%)	1 (4.5%)	1 (1.3%)
Side effects of oral treatment	N.A.	0 (0%)	0 (0%)
Renal	N.A.	0 (0%)	0 (0%)
Gastro-intestinal	N.A.	0 (0%)	0 (0%)
Hepatic	N.A.	0 (0%)	0 (0%)
Allergy	N.A.	0 (0%)	0 (0%)

N.A.: not applicable; ^a^: native valve disease (moderate–severe insufficiency/stenosis), only applicable for patients without prosthetic valves; ^b^: missing or inapplicable, n = 30; ^c^: missing or inapplicable, n = 25; ^d^: missing or inapplicable, n = 14.

**Table 3:** Assessment of hypothetical eligibility for IV–oral switch according to the original ESC guideline and the WikiGuideline criteria.

Criterion (Total n = 90)	ESC Criterion (n = 90)	Wiki Criterion (n = 90)
Left-sided endocarditis	79 (87.8%)	N.A.
Micro-organism (streptococci/*E. faecalis*)	85 (94.4%)	N.A.
Clinical course ^a^	36 (40.0%)	78 (86.7%) ^b^
CRP < 20 mg/L or decline to 25% of peak value Leukocytes < 15 × 10^9^/L Absence of fever >2 days	47 (52.2%) ^b^ 64 (71.1%) ^b^ 53 (58.9%) ^b^	N.A.
Clearance of bacteremia and antibiotic treatment duration ^c^	88 (97.8%) ^b^	87 (96.7%) ^b^
Echocardiogram (TEE) <2 days before switch and no indication for abscess or surgery	6 (6.7%) ^b^	N.A.
Echocardiogram (TEE/TTE) <7 days before switch and no indication for abscess or surgery	31 (34.5%) ^b^	N.A.
Oral antibiotic treatment susceptible/available (ESC: at least two antibiotics; Wiki: no specification)	83 (92.2%) ^b^	89 (98.9%)
Suspected reduced gastro-intestinal uptake (+BMI < 40 ESC)	2 (2.2%) ^b^	1 (1.1%) ^b^
Other indications for prolonged IV antibiotics (including socioeconomic determinants) ^d^	16 (17.7%) ^b^	16 (17.7%) ^b^

N.A.: not applicable; *E. faecalis*: *Enterococcus faecalis*; CRP: C-reactive protein; TEE: transesophageal echocardiogram; TTE: transthoracic echocardiogram; IV: intravenous. ^a^: See Table 1 for definitions; ^b^: missing data not included. Clinical course—Wiki, n = 4; CRP, n = 20; leukocytes, n = 21; fever, n = 27; clearance of bacteremia—ESC, n = 2; clearance of bacteremia—Wiki, n = 2; echocardiogram—ESC, n = 1; susceptibility antibiotics—ESC, n = 7. ^c^: ESC guidelines: >10 days of IV antibiotics/>7 days after surgery, see Table 1; ^d^: other indications include vascular graft involvement (n = 4, 4.4%), infected and unremoved CIED (n = 6, 6.7%), perivalvular complications (n = 4, 4.4%), the combination of perivalvular complications and vascular graft (n = 1, 1.1%) and other (n = 1, 1.1%).

## Data Availability

The data underlying this article will be shared upon reasonable request to the corresponding author.
